# Evolutionary and Genomic Diversity of True Polyploidy in Tetrapods

**DOI:** 10.3390/ani13061033

**Published:** 2023-03-12

**Authors:** Marcello Mezzasalma, Elvira Brunelli, Gaetano Odierna, Fabio Maria Guarino

**Affiliations:** 1 Department of Biology, Ecology and Earth Science, University of Calabria, Via P. Bucci 4/B, 87036 Rende, Italy; 2 Department of Biology, University of Naples Federico II, Via Cinthia 26, 80126 Naples, Italyfabio.guarino@unina.it (F.M.G.)

**Keywords:** chromosomes, evolution, genome, reproduction, vertebrates

## Abstract

**Simple Summary:**

Polyploidization, or whole-genome duplication (WGD), represents a dramatic event in evolution. Although its occurrence is much rarer in animals than in plants, distinct WGDs characterize the stem lineages of vertebrates and teleosts. In tetrapods, true polyploids have been described in all major groups and include a wide range of genomic configurations and modes of reproduction. In this work, we provide a comprehensive report on the presence of different types of polyploidy in tetrapods, with a particular focus on its genomic, evolutionary, and ecological diversity. We also describe the main routes of the formation of neopolyploids and discuss the two competing hypotheses that consider polyploidy either as a major force in evolution or, mainly, as an evolutionary dead end.

**Abstract:**

True polyploid organisms have more than two chromosome sets in their somatic and germline cells. Polyploidy is a major evolutionary force and has played a significant role in the early genomic evolution of plants, different invertebrate taxa, chordates, and teleosts. However, the contribution of polyploidy to the generation of new genomic, ecological, and species diversity in tetrapods has traditionally been underestimated. Indeed, polyploidy represents an important pathway of genomic evolution, occurring in most higher-taxa tetrapods and displaying a variety of different forms, genomic configurations, and biological implications. Herein, we report and discuss the available information on the different origins and evolutionary and ecological significance of true polyploidy in tetrapods. Among the main tetrapod lineages, modern amphibians have an unparalleled diversity of polyploids and, until recently, they were considered to be the only vertebrates with closely related diploid and polyploid bisexual species or populations. In reptiles, polyploidy was thought to be restricted to squamates and associated with parthenogenesis. In birds and mammals, true polyploidy has generally been considered absent (non-tolerated). These views are being changed due to an accumulation of new data, and the impact as well as the different evolutionary and ecological implications of polyploidy in tetrapods, deserve a broader evaluation.

## 1. Introduction

True polyploidy, or whole-genome duplication (WGD), is the genetic configuration wherein more than two sets of homologous chromosomes are present in the genome of both somatic and germline cells. A polyploidization event may result in instantaneous speciation by establishing reproductive barriers between the (neo)polyploid lineage and the parental species that lead to reproductive isolation in a single generation [1,2].

In higher plants, polyploidy is a widely recognized major evolutionary force. It is present in many species of numerous taxonomic groups and multiple, independent, WGDs characterized the early genomic evolution (paleopolyploidizations) of several evolutionary lineages [3,4].

In animals, true polyploidy has historically been overlooked, and its occurrence has been underestimated. It has been regarded as only marginally relevant in major processes of phylogenetic diversification and ecological adaptation and is generally considered incompatible with ontogenesis and sexual reproduction [5]. However, although true polyploidy is much rarer in animals than in plants, it is nevertheless present in all major vertebrate taxonomic groups. It occurs relatively frequently in some groups, and this may imply diverse mechanisms of origin, ploidy levels, and unique modes of reproduction [6,7].

It is now also widely accepted that at least two distinct ancient WGDs (traditionally described as the two-round hypothesis) occurred at the base of the evolution of vertebrates [8]. The first likely occurred after the advent of the urochordates, while the second occurred before the radiation of jawed vertebrates [9]. Furthermore, a third polyploidization event occurred in the stem lineage of teleosts (the teleost genome duplication—TGD) and additional rounds of WGDs occurred independently in different fish taxa (including Acipenseridae, Salmonidae, and Cyprinidae) [6,10].

In tetrapods, true polyploidy is known in all the major evolutionary lineages. It is relatively widespread in squamate reptiles, where it is usually associated with the insurgence of unisexual lineages, and is widely represented in amphibians, where it includes an unparalleled diversity of species and genomic configurations [6,11,12]. In mammals and birds, WGD was once thought to be absent, but it has been reported in octonotid species and parrots and chickens [13,14,15].

In this work, we describe the diverse routes through which neopolyploids can originate and provide an updated and comprehensive summary of the evolutionary and ecological diversity of polyploidy in all the major groups of tetrapods. We report the occurrence of different ploidy levels and related modes of reproduction and ecological adaptation, with a particular focus on the most notable cases. Finally, we compare and discuss the two major competing hypotheses that describe polyploidy as either a significant evolutionary advantage or as an evolutionary dead end.

## 2. Classification and Mechanisms of Polyploidy

**Figure 1:**
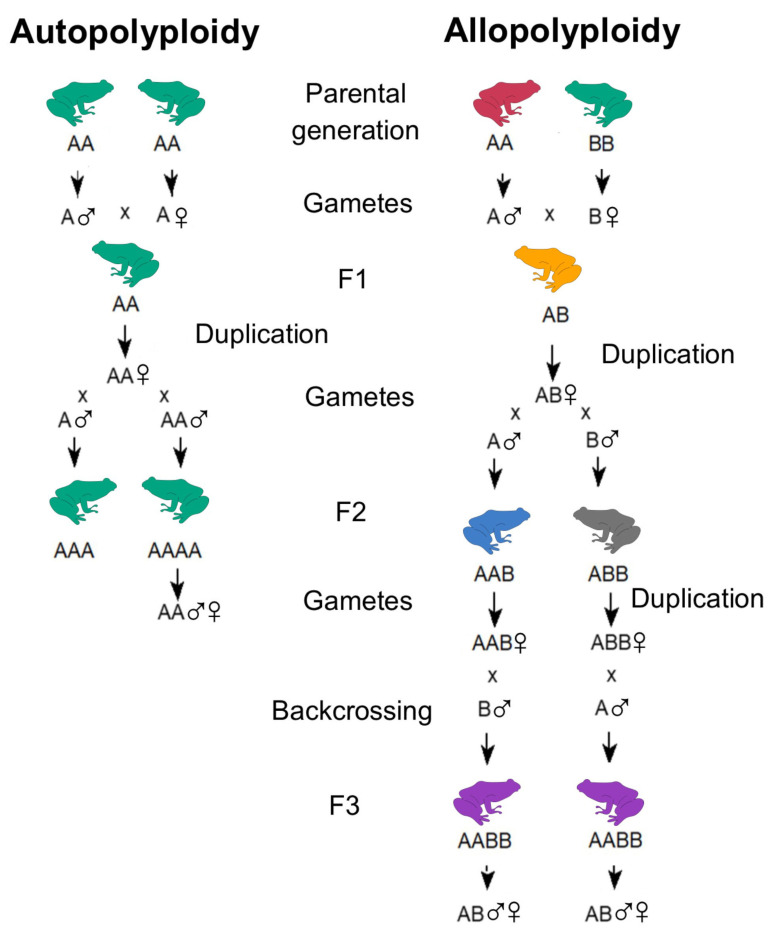
Schematic representation of autopolyploidy and allopolyploidy.

The two main pathways that may generate new polyploid lineages are known as autopolyploidy and allopolyploidy. The two pathways are distinguished based on whether the emergence of polyploidy occurs by means of the duplication of the chromosome set of a single species via mitotic (somatic doubling) or meiotic errors (gametic non-reduction) (autopolyploidy) or by means of the fusion of the chromosome sets of two different species followed by a WGD (allopolyploidy) [16,17,18] (Figure 1).

Both mechanisms played a significant role in the diversification of different tetrapod evolutionary lineages and indicate a wide range of different ecological and evolutionary implications, including the evolution of unisexual reproduction and the occurrence of speciation via hybridization [6,18].

In theory, autopolyploids may form within a single individual. However, the new polyploid lineage would likely suffer from heavy inbreeding depression and, in fact, most natural autopolyploids are generated after sexual reproduction [19,20].

As a result of their different origins, auto- and allopolyploidy can usually be detected using several molecular and cytogenetic techniques. For example, in autopolyploids, the chromosomes of a given quartet, sextet, or octet show complete sequence homology, indistinguishable banding patterns, and form multivalents in meiosis [21]. In allopolyploids, the two parental genomes may include sufficient differences to lead to the formation of highly homologous chromosome pairs and meiotic bivalents [21]. Although this typically results in the conservation of the two separate genomes, relatively higher levels of similarity between them correspond to higher chances for their homoeologs to pair, thus promoting exchanges of genetic material [22]. Furthermore, in allopolyploids, the mechanism of recombination between chromosomes from different sets, known as homoeologous exchange (HE), mostly involves regions of high similarity such as coding gene regions, thereby promoting the formation of novel genes and transcripts (neo- and subfunctionalization) [22].

An intermediate condition between auto- and allopolyploidy, known as segmental allopolyploidy, is characterized by the presence of two partially differentiated genomes, leading to the formation of either bivalents or multivalents [17,21]. For example, in allopolyploids, homoeologous exchange may generate mixed chromosomal patterns, where some regions maintain homoeologous regions while others appear homozygous by preferentially retaining one of the two parental genomes and showing an autopolyploid-like structure [23]. Over generations, populations with homoeologous exchange may present highly variable individuals at the chromosomal level, which are differentially affected by natural selection [24].

Regardless of how it originates, the emergence of polyploidy presents several challenges for cell processes, physiology, and genome stability [25,26]. The success of neopolyploids is ultimately determined by survival and reproduction rates, competition with parental lineages, and the complex, long-term ecological and evolutionary consequences of polyploidy [27]. New polyploids that are incapable of overcoming the early phase of genomic instability are usually heavily penalized by selection, while those capable of adapting to the initial genome shock may form a new polyploid population or species (neopolyploids) [28]. In neopolyploids, renewed genomic stability may be achieved through a process called diploidization, which involves the progressive differentiation (or loss) of duplicate genetic material (repetitive DNA, genes, and whole chromosomes), which, ultimately, restores a diploid genome structure [29]. In diploidization, the deletion of DNA repeats is typically coupled with the neo- and subfunctionalization of duplicated genes and chromosome rearrangements, eventually leading to the formation of a functionally pseudodiploid genome [29].

## 3. Amphibians

No other tetrapod group exhibits a comparable number of polyploid taxa or a similar variety of different polyploid genomic configurations and modes of reproduction as the modern amphibians [6,11,30]. This is likely due to a combination of several factors, including a high level of genomic plasticity and the occurrence of undifferentiated sex chromosomes that do not require dosage compensation [11,31].

**Figure 2:**
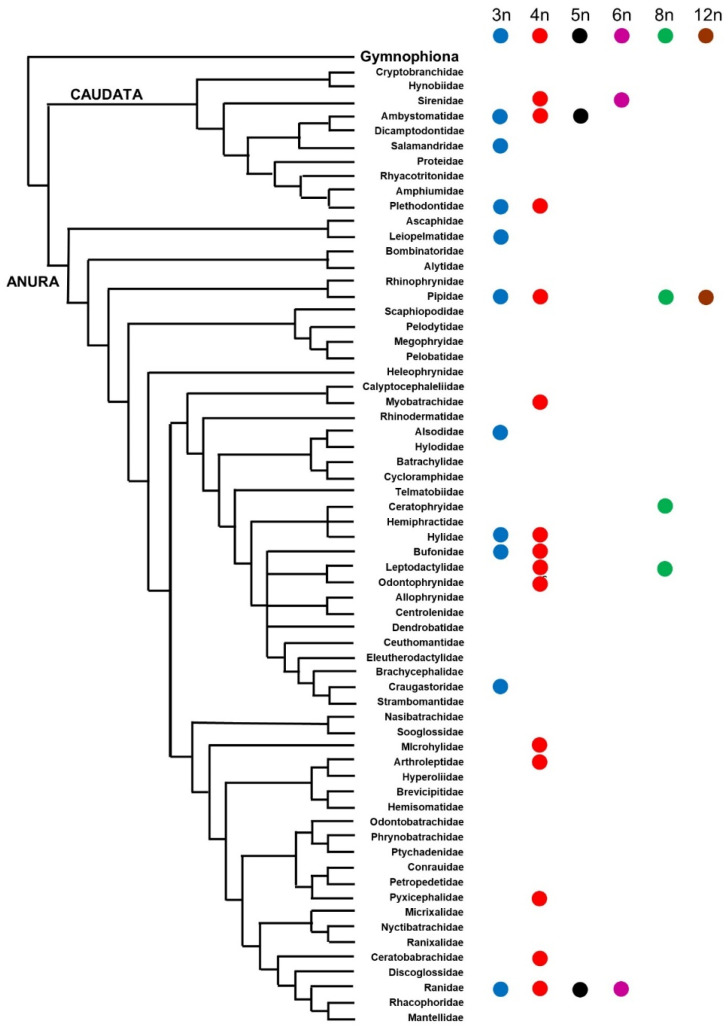
Phylogenetic distribution of polyploidy in Amphibia. Phylogenetic relationships redrawn from [34] and based on different datasets [35,36,37,38,39].

To date, polyploidy in amphibians is known to occur in more than 100 species distributed across 19 families of Urodela (4 families) and Anura (15 families), while it has not yet been found in Gymnophiona, possibly as a result of the low number of caecilian species that have been studied with cytogenetic methods [11,32,33] (Figure 2).

Natural (not experimentally induced) polyploidy in amphibians ranges from 3n (in 10 different families) to 12n (only in the genus *Xenopus*, family Pipidae) [11,32,33] (Figure 2; phylogenetic relationships redrawn from AmphibiaWeb [34] and based on the datasets by Blackburn and Wake, Feng et al., Jetz and Pyron, Streicher et al., and Yuan et al.) [35,36,37,38,39]. Excluding diploids, tetraploidy (4n) is the most phylogenetically widespread ploidy level in amphibians, occurring in 14 different families. Pentaploidy (5n) and hexaploidy (6n) are known in two families, respectively, while octaploidy (8n) has been documented in Pipidae, Ceratophryidae, and Ranidae [11,32,33], (Figure 2). In general, amphibian triploids, tetraploids, and octaploids have all been hypothesized to occur either via auto- or allopolyploidy, while hexaploids and dodecaploids are mostly known to occur via allopolyploidy [6,30,40].

In amphibians, the genomic, ecological, and evolutionary innovations introduced by polyploidy have often led to phylogenetic diversification and cladogenesis, and several striking cases concern different genera that appear to be particularly prone to recurrent WGDs [6,11].

In the North American mole salamanders of the genus *Ambystoma*, polyploidy is associated with a peculiar evolutionary pathway that has led to the diversification of a unique reproductive mechanism known as kleptogenesis. In particular, *Ambystoma* is composed of several bisexual and unisexual species and populations (from 2n to 5n) [41]. Unisexual *Ambystoma* often live in association with one or more bisexual species, which act as sperm donors [41]. Apparently, unisexuality follows a classic gynogenetic pathway in *Ambystoma*, where male gametes activate embryonic development, but the paternal DNA is not incorporated in the offspring [42]. However, in some cases, male gametes fuse with eggs in a manner similar to sexual reproduction or they may substitute one of the haploid genomes of the female [41,43]. This may lead to multiple paternity and/or the elevation of the ploidy of the zygote [37,44]. These mechanisms may favor the genetic variability of this bisexual/unisexual species complex and provide new adaptive advantages. For example, a recent study highlighted the occurrence of a higher tissue regeneration rate in polyploid mole salamanders compared to congeneric diploid species [45].

Among the other polyploid Urodela, many species (e.g., *Eurycea bilineata*, *Cynops pyrrhogaster*, *Lissotriton alpestris,* and *Notophthalmus viridescens*) include naturally occurring autopolyploid individuals found in mosaic populations along with normal diploids [46,47,48,49].

In the Eurasian toads of the genus *Bufotes*, diploid, triploid, and tetraploid populations represent a complex of more than 10 distinct haplogroups with partial range overlaps [50,51]. Molecular and cytogenetic analyses suggest that diploid species reproduce bisexually and that the 4n *B. pewzowi* likely represent an allopolyploid species [51]. Interestingly, triploids can originate via hybridization between 2n and 4n populations, but the 3n *B. baturae* reproduce sexually through a unique system of differential meiosis, leading to the fusion of haploid sperm and diploid eggs [51,52].

Another interesting example of polyploidy is the green frog *Pelophylax* kl. *esculentus* (Ranidae), which represents a hybrid between *P. lessonae* (LL genome) and *P. ridibundus* (RR genome) (two diploid bisexual species). In this complex, diploid (LR genome) and polyploid hybrids (from 3n to 5n) may occur in sympatry [53]. However, the reproduction of diploid (LR) hybrids relies on the parental species. In fact, in the *lessonae-esculentus* system, LR hybrids produce only R gametes and must mate with *P. lessonae* in order to generate new hybrids. Conversely, in the *ridibundus*-*esculentus* system, LR hybrids mostly produce L gametes and must mate with *P. ridibundus* to generate new hybrids [53].

Two notable but different examples of recurrent polyploidy in Amphibia are represented by the genera *Neobatrachus* (Lymnodynastidae) and *Xenopus* (Pipidae). At least five diploid and four tetraploid *Neobatrachus* species occur in Australia. The tetraploid species are likely all autopolyploids, which can backcross with syntopic diploids, producing triploid hybrids [54]. In *Xenopus*, there are at least 28 known species with different polyploidy levels (3n, 4n, 8n, and 12n), thus representing the greatest intrageneric variability in ploidy among vertebrates [11,55]. Polyploid *Xenopus* species likely originated via multiple, independent events of allopolyploidization. In particular, tetraploidy arose at least twice, octaploidy at least three times, and dodecaploidy at least four times, independently [11,55].

Besides naturally occurring WGD, there are several physical conditions that can experimentally induce polyploidy (triploidy, tetraploidy, and pentaploidy) and aneuploidy in both Urodela and Anura, including temperature shock and hydrostatic egg compression [11,33]. The cellular mechanisms that produce induced polyploids are still unclear, but these findings further highlight the peculiar genomic plasticity of amphibians and their predisposition to WGD.

## 4. Reptiles

**Figure 3:**
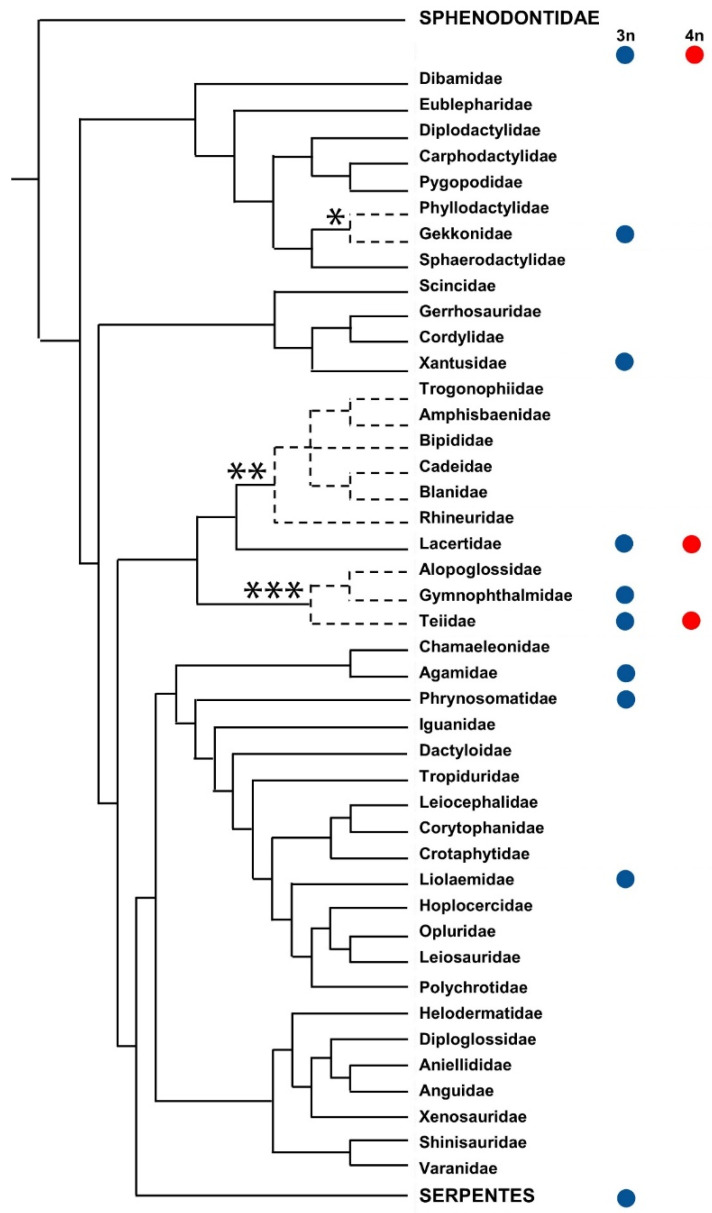
Phylogenetic distribution of polyploidy in squamates. Phylogenetic relationships redrawn from [67]. Dashed lines represent phylogenetic relationships determined by * Gamble et al. [68], ** Vidal and Hedges [69], and *** Hernández-Morales [70].

Reptiles are emerging model organisms in the study of genomic diversification, chromosome evolution, and sex determination [56,57,58,59,60,61] and represent an interesting group whose study facilitates a better understanding of the particular evolutionary pathways of polyploidy in amniotes. In reptiles, polyploidy is mostly present in squamates (particularly in lizards) in the form of triploids (Figure 3), which are usually associated with hybridization and parthenogenesis. Furthermore, squamates represent the only vertebrates with obligate or true (sperm-independent) parthenogenesis [12,62,63], which can also be characterized by occasional backcrossing with one or more parental or related species [64]. Nevertheless, unisexual reproduction is not restricted to polyploid squamates, and facultative parthenogenesis is generally associated with diploid species [65,66].

In squamate phylogeny [67,68,69,70], polyploidy has been documented in eight lizard families (Gekkonidae, Xantusidae, Lacertidae, Gymnophthalmidae, Teiidae, Agamidae, Phrynosomatidae, and Liolaemidae) (Figure 3) (e.g., see [6,12]). However, the total number of currently available karyotypes represent only about 5% of the currently described lizard species [71], and true polyploidy might also be present in other cytogenetically understudied taxonomic groups. In addition, polyploid and parthenogenetic lizard lineages are not uniformly distributed among different taxa, and some families (e.g., Lacertidae and Teidae) and genera (e.g., *Darevskia* and *Aspidoscelis*) show a relatively higher number of clades with a polyploid genomic configuration and unisexual reproduction [12]. 

Squamate polyploidy is generally related to events of speciation through hybridization that can generate allopolyploid lineages and mosaic populations (see e.g., [6,72]). Furthermore, the phylogenetic and genetic complexity of some polyploid lizard clades is increased by reticulate evolution and secondary hybridization [6]. For example, the rock lizard *Darevskia unisexualis* (Lacertidae) is a diploid parthenogenetic species that originated from hybridization between *D. raddei nairensis* and *D. valentine* [73]. The females of *D. unisexualis* can mate with males of the parental *D. valentini*, giving rise to triploid and tetraploid secondary hybrids, including sterile individuals, intersexes, and fertile males and females [74]. 

Another striking example of reticulate evolution, and the first case of hybrid tetraploid individuals in lizards, was reported in *Aspidoscelis* (*Cnemidophorus*) (Teidae), which likely represents the result of a secondary hybridization between the sympatric *A. inornatus* (a diploid species that reproduces sexually) and *A. exanguis* (an allotriploid, hybridogenetic, parthenogenetic species) [42]. In recent years, hybridization between *A. inornatus* and *A. exanguis* was performed in the laboratory, leading to the generation of a self-sustaining lineage of clonal tetraploid females [75]. The existence of natural tetraploid hybrid populations in *Aspidoscelis* remains to be confirmed, but the evidence provided by [75] represents the first documented case of a tetraploid parthenogenetic amniote. 

In snakes, true polyploidy is only known in two species. The brahminy blind snake *Indotyphlops braminus* (Typhlopidae) shows a triploid genomic configuration (3n = 42) [76,77,78] with all-female populations and represents the only known snake species to reproduce via obligate parthenogenesis. 

In the generally diploid *Elaphe maculata* (with a ZW sex chromosome system) (Colubridae), the spontaneous occurrence of triploid male individuals has been reported by the authors of [79]. Both ZZ and ZZW individuals of *E. maculata* develop male gonads, leading to the hypothesis that in this species (and possibly in other squamates), the ratio between the number of Z chromosomes and autosomes (not the presence of the W chromosome) controls sex determination [79]. In fact, although most squamate species are characterized by genetic sex determination with either male (XY) or female heterogamety (ZW), no single sex-determining gene has been unambiguously isolated so far [71,80], and new insights into squamate sex determination may stem from future studies of polyploid lineages.

In non-squamate reptiles, the only case reported so far, and a noteworthy example of the plasticity of polyploidy in tetrapods, is represented by the twist-necked turtle *Platemys platycephala* (Chelidae). In this species, a peculiar form of diploid–triploid mosaicism was first recorded in natural populations [81], but while specimens of *P. platycephala* from Suriname and French Guiana exhibit various levels of ploidy (including diploids, triploids, diploid–triploid mosaic individuals, and triploid–tetraploid mosaic individuals) [81,82], populations from Brazil and Bolivia show only diploid individuals [81,83]. It was also hypothesized that a peculiar subsexual mode of reproduction occurs in this species, with females reproducing parthenogenetically (or gynogenetically), while males are generated by a temperature-dependent form of sex determination [84]. However, analyses of gonadal tissues indicated that normal (haploid) gametes were produced in males irrespective of the ploidy level of somatic tissues, thus supporting the occurrence of sexual reproduction and discarding the notion of subsexual or unisexual reproduction in this species [82]. Furthermore, there are no known heteromorphic sex chromosomes in *P. platycephala,* and sex determination has been hypothesized to be driven by temperature-dependent mechanisms or ploidy levels [85]. In fact, different ploidy levels in *P. platycephala* are statistically associated with sex, with triploidy being largely predominant in males [85]. Therefore, it appears that the different ploidy levels do not constitute an evolutionary constraint toward unisexual reproduction in *P. platycephala* but might represent a genomic driver for the evolution of new mechanisms of sex determination.

## 5. Birds

Two different cases of true polyploidy are known in birds. In *Gallus domesticus* (Galliformes), triploid and tetraploid individuals are known to appear with low frequency, showing intersex characteristics [86,87]. Intersex polyploids in *G. domesticus* (usually triploids) may develop either parthenogenetically or from fertilization between reduced and unreduced gametes [86].

The other known case concerns the blue-and-yellow macaw, *Ara ararauna* (Psittaciformes), where triploidy was cytogenetically detected by Tiersch et al. [13] in one individual. The occurrence of true polyploidy in birds is thus currently considered a genetic abnormality, limited to individuals and mostly produced by meiotic errors [88]. As in the case of mammals (see below), polyploidy has traditionally been considered to be suppressed in birds mostly because of its negative effects on development and dosage compensation [6,89]. Nevertheless, the tolerance to genome doubling as well as the occurrence of parthenogenetic individuals suggest the possibility of a wider presence of true polyploidy in different bird clades, perhaps representing undetected evolutionary scenarios in poorly studied taxonomic groups. 

## 6. Mammals

Rare cases of polyploidy have been documented in mammals, and almost all of them result in non-vital embryos or prenatal death [90]. For example, in humans, polyploidy may occur as triploid (3n = 69) or tetraploid (4n = 92) embryos, which are generally formed by polyspermy or abnormal chromosome segregation either via diandry (an extra haploid set from the father) or digyny (an extra haploid set from the mother) and typically represent non-vital conditions or lead to abnormal development [28,91].

As a consequence of most empirical evidence gathered on several negative effects of mammalian polyploidy on sex determination systems, development, and the mechanisms of dosage compensation, true polyploidy was considered non-existent in mammals [6,89,92] until the discovery of tetraploidy in the red viscacha rat (*Tympanoctomys barrerae*) (4n = 102) (Octodontidae) [14]. This finding was later questioned by the localization of only one NOR-bearing chromosome pair in the karyotype of the species and the occurrence of just two clear hybridization signals using different chromosome-specific probes [93]. These results were interpreted as proof of the occurrence of only two copies of each chromosome in the genome of *T. barrerae*, which was consequently regarded as a diploid species [93]. However, the occurrence of tetraploidy was eventually confirmed in *T. barrerae* by further analyses with molecular cytogenetics, which produced differential results on diploid and tetraploid octodontid species [94]. On the other hand, the localization of the loci of NORs on only two homologous chromosomes in the karyotype of *T. barrerae* is likely due to nuclear dominance, a quite well-known regulatory mechanism in allopolyploids and diploid hybrids [95,96]. In fact, the diploid-like meiotic behavior, heteromorphic G-bands, and the chromosomal variances detected among different individuals of *T. barrerae* were all considered indicators of allopolyploidy [14,94,97].

Interestingly, a second case of tetraploidy in Octodontidae was discovered in the golden viscacha rat *Pipanacoctomys aureus* (4n = 96) [97]. Phylogenetic relationships among Octodontidae support the sister clade relationship between *T. barrerae* and *P. aureus,* and the current hypotheses suggest that hybridization between a common ancestor of the two species and a species showing a similar karyotype to that of *Octomys mimax* (2n = 56), followed by WGD, represents a likely scenario for the origin of tetraploidy in the group [94,98]. On the other hand, *T. barrerae* and *P. aureus* remain the only known polyploid mammal species, and future research should further explore the possible contribution of WGD to the evolutionary diversification of other mammalian clades.

## 7. Advantages and Disadvantages of Polyploidy

Two main opposite hypotheses have been traditionally invoked in the ongoing debate on the evolutionary implications of polyploidy. In fact, the peculiar developmental and genetic conditions arising with polyploidy can be either beneficial or deleterious for a given lineage.

The first hypothesis perceives polyploidy as a significant evolutionary force, providing neopolyploids with new characteristics that can offer several selective advantages over parental and phylogenetically closely related lineages [18]. Overall, the widespread occurrence of polyploidy among animals and plants and the WGDs at the base of many evolutionary lineages have been considered measures of its evolutionary success [99,100]. 

In general, most of the possible advantages of polyploidy are directly or indirectly linked to the establishment of higher degrees of gene diversity and heterozygosity, the potential loss of self-incompatibility, and the insurgence of asexual reproduction [18]. Hybrid and polyploid genomes may bring genetic (and phenotypic) novelties through neo- or subfunctionalisation, leading to the acquisition of new adaptations [18,101]. Heterosis (hybrid vigor) and gene doubling may also mitigate the effects of deleterious mutations and genotoxicity, providing competitive advantages to new polyploid generations [102,103]. In plants, allopolyploidy is frequently related to the appearance of invasive species and, in different animal clades, WGD provides new ecological solutions in stressful environmental conditions [104,105,106]. For example, a recent study on three animal clades (Amphibia, Actinopterygii, and Insecta) showed a positive association between the occurrence of polyploid lineages and latitude, with glaciations representing the strongest ecological driver of polyploidy in animals [107].

The second, opposing hypothesis recognizes polyploidy mostly as an evolutionary dead-end. This view was initially proposed by Stebbins [108,109] and Wagner [110], who described polyploidy as an essentially detrimental condition with a marginal contribution in large-scale scenarios. In fact, even considering the WGDs at the base of different evolutionary lineages, true polyploid species are rare in animals [5,6] because most neopolyploids are likely unable to overcome the initial bottleneck of genomic instability [20,111,112]. New polyploid lineages reportedly have lower rates of speciation and higher extinction rates than diploids, and various studies indicate that most neopolyploid lineages might disappear in a few generations [2,113].

Among the outcomes that have been associated with polyploidy, there are several negative effects on the general cellular structure that are related to the increase in the DNA content and cell dimensions, with possible disruptive consequences on intracellular functions [114]. Cell division in polyploids can be negatively affected by mitotic and meiotic instability, leading to the formation of aneuploid cells [115]. In meiosis, aneuploidy can be a result of the formation of multivalents in autopolyploids [116] and the problematic pairing of homeologs in allopolyploids, which are processes requiring specific mechanisms to sort subgenomes [117].

Genome doubling may also negatively affect gene expression and generate epigenetic instability [1,118]. The severity of such effects may vary in different lineages, but a new equilibrium must be reached in neopolyploids through genome reorganization and the regulation of gene expression (e.g., via diploidization and DNA methylation) [119,120].

The two major theories proposing polyploidy as either an evolutionary force or dead-end present different strengths and weaknesses, and neither of them can be seen as universally correct or incorrect within a large taxonomic group. In fact, the evolutionary success of polyploidy should be interpreted as a lineage-specific outcome, directly and indirectly linked to multiple, complex factors. Among them, the particular genomic configuration, the type of polyploidy (auto- or allopolyploidy), environmental conditions, and the possible co-occurrence of different non-polyploid related species may all play a significant role in determining the evolutionary success of a given neopolyploid lineage [27,104,121].

This view also seems to be supported by the recurrent polyploidy in some taxa (e.g., in *Xenopus*, in *Bufotes* among Amphibia, and in *Darevskia* and *Aspidoscelis* among squamates) [12], which might imply particular genomic predispositions to WGD and/or ecological conditions favoring neopolyploids. Moreover, polyploids (with the same or different ploidy levels) with different origins can be genetically and/or ecologically dissimilar even in closely related lineages, as in the case of homoeologous exchange [23,122], presenting distinct advantages or disadvantages in different environmental conditions.

## 8. Conclusions and Prospects

Polyploidy is a natural pathway of genomic evolution in most higher tetrapod taxa with a considerable number of different chromosomal configurations and evolutionary and ecological innovations. However, polyploidy is not evenly distributed in the major tetrapod groups. It is widely represented in amphibians and reptiles, particularly in some genera, where recurrent WGDs have led to multiple speciation events, diploid/polyploid species complexes, and the appearance of unique modes of reproduction. Modern amphibians present more than 100 auto- and allopolyploid species (from 3n to 12n) of Anura (in 15 familes) and Urodela (4 families), while WGD has not been reported yet in Gymnophiona. In reptiles, polyploidy (triploidy and tetraploidy) has mostly been documented in squamates (in eight families), where it is usually associated with speciation by hybridization, parthenogenesis, and, in some cases, reticulate evolution and secondary hybridization. In mammals, two tetraploid species have been reported in the family Octodontidae, while in birds, WGD has been found in individuals of the Galliformes and Psittaciformes orders.

Overall, polyploidy has been considered as either a major force in evolution or as an evolutionary dead-end. However, the evolutionary success of polyploidy should be treated as a lineage-specific outcome linked to multiple factors, including the particular genomic configuration, environmental conditions, and the possible co-occurrence of different, non-polyploid, related species.

Although recent studies greatly improved the knowledge regarding the evolutionary contribution of polyploidy in tetrapods, its occurrence is likely still underestimated. Future research should focus on understudied taxonomic groups in order to better describe the diversity and the phylogenetic distribution of polyploidy in tetrapods. Furthermore, the implementation of modern multidisciplinary approaches, including a combination of molecular, cytogenetic, and bioinformatic techniques, will provide the opportunity to explain still unclear genetic and developmental mechanisms of the formation of neopolyploids, modes of reproduction, and sex determination.

## Data Availability

Not applicable.
